# Routine Surveillance of Healthcare-Associated Infections Misses a Significant Proportion of Invasive Aspergillosis in Patients with Severe COVID-19

**DOI:** 10.3390/jof8030273

**Published:** 2022-03-08

**Authors:** Julia Ebner, Miriam Van den Nest, Lukas Bouvier-Azula, Astrid Füszl, Cornelia Gabler, Birgit Willinger, Magda Diab-Elschahawi, Elisabeth Presterl

**Affiliations:** 1 Department of Infection Control and Hospital Epidemiology, Medical University of Vienna, Waehringer Guertel 18-20, 1090 Vienna, Austria; julia.ebner@meduniwien.ac.at (J.E.); miriam.vandennest@meduniwien.ac.at (M.V.d.N.); lukas.bouvier-azula@meduniwien.ac.at (L.B.-A.); astrid.fueszl@meduniwien.ac.at (A.F.); magda.diab-elschahawi@meduniwien.ac.at (M.D.-E.); 2 Research Documentation and Analysis, IT4Science, Medical University of Vienna, Spitalgasse 23, 1090 Vienna, Austria; cornelia.gabler@meduniwien.ac.at; 3 Division of Clinical Microbiology, Department of Laboratory Medicine, Medical University of Vienna, Waehringer Guertel 18-20, 1090 Vienna, Austria; birgit.willinger@meduniwien.ac.at

**Keywords:** COVID-19, superinfection, invasive aspergillosis, surveillance, intensive care

## Abstract

Rates of invasive aspergillosis (IA) among COVID-19 ICU patients seem to reach over 30% in certain settings. At Vienna General Hospital (VGH), all rooms in COVID-19 ICUs were put under negative pressure as a protective measure, thus increasing the risk of exposure to environmental pathogens for patients. Even though all ICU patients are surveilled for healthcare-associated infections (HAI), there were concerns that the routine protocol might not be sufficient for IA detection. We reviewed the electronic patient charts of all patients with COVID-19 admitted to ICUs between 1 March 2020 and 31 July 2021 for fungal co- or superinfections, comparing four diagnostic algorithms based on different recommendations for the diagnosis of IA (according to EORTC/MSG, BM-AspICU, IAPA and CAPA) to our routine surveillance protocol. We found that out of 252 patients who were admitted to the ICU during the study period, 25 (9.9%) fulfilled the criteria of probable or possible IA of at least one algorithm. The IAPA definitions detected 25 and the CAPA definition 23 probable and 2 possible cases, out of which only 16 were classified as hospital-acquired IA by routine surveillance. In conclusion, adjustment of the routine protocol using a classification system especially designed for respiratory viral illness seems useful for the surveillance of IA in a highly vulnerable patient cohort.

## 1. Introduction

Invasive aspergillosis (IA), which is most commonly caused by *Aspergillus fumigatus* [1,2,3], is a well-known complication in patients with hematological malignancies or bone marrow transplantation due to severe neutropenia [4,5]. It also occurs in patients with severe respiratory conditions needing high-dose corticosteroid or other immunomodulating treatment [3]. IA is associated with mortality rates well above 50% in ventilated patients in the ICU [1,2].

Although reports on IA rates among COVID-19 ICU patients vary, some authors have reported rates of over 30% [6,7]. These numbers are high when compared with non-COVID ICU patients, where incidence was estimated at 0.3 to 5.8% [8,9]. Several factors seem to lead to the increased susceptibility of COVID-19 patients to IA, i.e., ARDS, prolonged hospital/ICU stay, prolonged mechanical ventilation and/or extracorporeal membrane oxygenation (ECMO) and therapy with corticosteroids [10]. The Centers for Disease Prevention and Control (CDC) recommend increased awareness regarding IA in patients with severe COVID-19, particularly in those who remain febrile even after a course of adequate antibiotic treatment against bacterial co-infections [11].

Diagnosis of IA is still a challenge despite considerable advances in the last few decades. Diagnosis is mainly based on cultural techniques, histopathology and biomarkers. It is additionally supported by typical radiological signs and by the presence of pre-existing conditions that increase susceptibility to IA.

In COVID-19 ICU patients, however, diagnosis of IA is particularly challenging due to COVID-19-damaged lungs and the associated lesions in CT scans masking IA. It is further hampered by the reluctance to obtain invasive or bronchoscopic specimens from the lower respiratory tracts of highly infectious patients [12,13]. Colonization of the respiratory tract with filamentous fungi, ubiquitously present in the ambient air, may occur. Hence, the interpretation of these results can be challenging and experts have expressed worries of overtreatment due to overly sensitive diagnostic criteria [14].

At VGH, patient rooms in ICU units are usually designed to function as a protective environment. They have a ventilation system equipped with high-efficiency particulate air (HEPA) or even ultra-efficiency particulate air (ULPA) filters and relative positive room pressure to reduce the exposure to particles and spores. In contrast, at the beginning of the pandemic, rooms in ICUs that treated COVID-19 patients were converted into makeshift airborne infection isolation rooms (AIIR) that lacked anterooms but offered certain protection to staff and other patients through reversed airflow and relative negative pressure [15]. However, the reversed airflow direction may increase the risk of exposure to airborne environmental pathogens such as fungal spores for ICU patients themselves.

**Figure 1:**
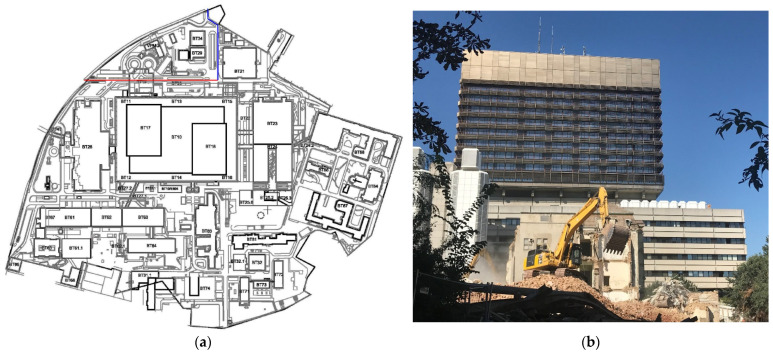
(**a**) Ground plan of VGH, (**b**) photo of demolition site, BT = building.

Additionally, from July until October 2020, demolition of one of the oldest buildings of VGH (Figure 1, BT83) was inevitable and sparked worries of dust accumulation despite concomitant dust-minimizing measures. This may have further increased the risk of IA in our particular setting as the demolition and reconstruction of buildings is known to be associated with increased incidence [16].

Even though all ICU patients at VGH are under continuous surveillance for healthcare-associated infections (HAI) according to the European Centers of Disease Prevention and Control (ECDC) HAI-Net ICU protocol (including different types of pneumonia) [17], we were concerned that this protocol alone might not be sufficient for IA detection as it is mainly focused on bacterial infections.

We conducted an observational study on the incidence of IA in COVID-19 patients admitted to the ICU and retrospectively compared four diagnostic algorithms based on different recommendations for the diagnosis of IA as an add-on to our routine surveillance protocol.

## 2. Materials and Methods

### 2.1. Study Setting

Vienna General Hospital is a 1740-bed academic hospital providing treatment for 347,926 outpatients and 59,454 inpatients in 2020. There are 15 intensive care units with 134 beds at the hospital, comprising three surgical units, one cardiothoracic surgery unit, one vascular surgery unit, one neurosurgical unit, one burn unit, one transplant unit, three internal medicine units, three pediatric units and one psychiatric unit. During the COVID-19 pandemic, VGH mainly served as a reference center for patients with severe COVID-19, requiring intensive care including extracorporeal membrane oxygenation (ECMO). During the first national lockdown in the spring of 2020, all non-urgent patient care at VGH was postponed in order to avoid an overload of ICUs and normal wards alike. By summer 2020, routine medical operation was gradually resumed.

We retrospectively analyzed data of SARS-CoV-2 intensive care patients with filamentous fungal co- and superinfections at a tertiary care center. The study was approved by the Medical University of Vienna’s ethics committee (EK Nr. 1388/2021).

### 2.2. Hospital Ventilation and Air Conditioning (HVAC) System

VGH’s HVAC system for the ICUs uses 100% fresh air. The ventilation apertures are situated on the 12th floor of the main hospital building (Figure 1, BT10) between the two towers (BT 17/18) that accommodate the hospital wards. The HVAC system of ICU patient rooms has three filter stages (ISO ePM1 > 50%/ePM1 > 80%/EN1822 H13 compact filter) and provides HEPA filtered air to the highly vulnerable patients.

Air change rates in the patient rooms range from 10 to 26 per hour, with the highest air change rates achieved on the burn unit and units that care for patients immediately after organ transplantation.

All ICU rooms are designed as protective environment rooms, where the airflow creates a mild positive differential pressure of 10 to 15 Pa with respect to adjacent rooms in order to protect vulnerable individuals from airborne pathogens. In the early stages of the COVID-19 pandemic, the airflow at ICUs caring for COVID-19 patients was adjusted so that the resulting negative differential pressure would protect HCW and fellow patients from aerosols dispersed by COVID-19 patients.

### 2.3. Patients

All patients admitted to ICUs due to severe COVID-19 between 1 March 2020 and 31 July 2021 were included in the study. Electronic patient charts of all identified patients were reviewed in order to establish the diagnosis of a fungal co- or superinfection. Apart from information on fungal infection, we gathered data on laboratory results (creatinine, CRP, hemoglobin, leukocytes and thrombocytes on ICU admission), length of stay (LOS), ventilation and outcome. For patients with IA, data on severity of disease (SAPS score), hospital stay (length of stay, origin of patient, outcome), SARS-CoV-2 infection (including ECMO and intubation) and medication (antimycotics, corticosteroids, IL-6 inhibitors) were collected additionally.

### 2.4. Diagnostic Criteria

Since 2017, routine surveillance of healthcare-associated infections for ICU patients has been in place at 14 out of 15 ICUs at VGH. Surveillance is conducted using the ECDC HAI-Net ICU protocol, version 2.2 [17]. All patients admitted to the ICU for at least 48 h are included; HAI diagnoses are primarily based on sites of infection and include, among others, pneumonia, blood stream infection, catheter-associated infection or urinary tract infection. Diagnosis of pneumonia is divided into five hierarchically structured subcategories (PN1-5), depending on microbiological and clinical evidence. For all definitions of pneumonia, patients must show radiological and clinical signs (fever or leukopenia/leukocytosis and sputum production, cough/dyspnea, suggestive auscultation or worsening gas exchange). PN1 and PN2 focus on positive quantitative bacterial culture from minimally or possibly contaminated material. Code PN3 is relevant for fungal infections, as it is designed for alternative microbiology methods. It includes positive exams for viral pneumonia or pneumonia caused by particular germs such as fungi. In contrast to PN1–3, PN4 and PN5 require at least one additional clinical sign for diagnosis; PN4 is for patients with positive sputum culture or non-quantitative LRT specimen culture, and PN5 for clinical pneumonia only.

In order to evaluate the performance of the HAI-Net ICU protocol for IA in COVID-19 patients, we compared it to the diagnostic criteria defined by the following publications: the Consensus Definitions of Invasive Fungal Disease From the European Organization for Research and Treatment of Cancer and the Mycoses Study Group Education and Research Consortium (EORTC/MSG); the modified version for diagnosing invasive pulmonary aspergillosis in critically ill patients (BM-AspICU) [18]; the definition for influenza-associated pulmonary aspergillosis in ICU patients (IAPA) [19] and the COVID-19-associated pulmonary aspergillosis 2020 ECMM/ISHAM consensus criteria for research and clinical guidance (CAPA) [20].

All algorithms require culture from sterile material or histopathology for the classification of proven cases, whereas they differ regarding the patients’ underlying diseases that are accepted as host factors for probable IA. According to EORTC/MSG, a probable case of IA requires at least one host factor reflecting severe immunosuppression (severe neutropenia < 0.500, hematologic malignancy, receipt of an allogeneic stem cell or solid organ transplant, prolonged use of corticosteroids, treatment with T-cell or B-cell immunosuppressants, inherited severe immunodeficiency, acute graft-versus-host disease grade III or IV), which are not generally present in ICU patients. BM-AspICU is designed for ICU patients and uses chronic diseases such as chronic obstructive pulmonary disease (COPD), viral respiratory diseases including SARS-CoV2 infection, hepatic insufficiency, diabetes, chronic alcohol abuse and other chronic diseases in addition to the EORTC/MSG host factors. IAPA focuses on influenza patients; therefore, a positive influenza PCR or antigen test in patients presenting with influenza-like illness is the only host factor (for the purpose of our study, we substituted this with SARS-CoV-2 PCR). Correspondingly, according to CAPA, only patients with COVID-19 needing intensive care are included. CAPA also allows classification of possible in addition to probable cases when culture, PCR or antigen tests are positive in non-bronchoscopically obtained specimens only.

For probable or possible IA, all algorithms additionally require some form of clinical or radiological sign and a mycological sign (either culture, antigen assay and/or PCR). Main features are shown in Table 1; detailed criteria are displayed in Supplementary Material File S1.

### 2.5. Diagnostic Methods

Mechanically ventilated patients were routinely screened for filamentous fungal infection using culture and galactomannan assay out of bronchoalveolar lavage upon intubation. In case of clinical deterioration, galactomannan assay out of respiratory specimens or serum was repeated and β-D-Glucan assay out of serum was conducted. The Division of Clinical Microbiology at VGH further processed respiratory specimens using standard microbiologic methods. For cultural detection of fungi, specimens were set onto Sabouraud Dextrose agar, CHROMagarCandida^®^, Brain-Heart Infusion Agar slants and Sabouraud Glucose broth (all Becton Dickinson, Heidelberg, Germany) and incubated at both 35–37 °C and 28–30 °C for up to three weeks. Upon cultural growth, fungi were further identified by macroscopic and microscopic assessment and MALDI-TOF (Bruker, Billerica, MA, USA). Resistance testing was performed using E-test^®^ on RPMI agar. Minimal inhibitory concentrations (MIC) were interpreted according to the clinical breakpoints issued by the European Committee on Antimicrobial Susceptibility Testing (EUCAST). For antigen detection, Platelia™ Aspergillus Ag (Bio-Rad, Basel, Switzerland) and Fungitell^®^ Assay (East Falmouth, MA, USA) were used for aspergillus-galactomannan (serum, BAL) and (1-3)-β-D-Glucan (serum), respectively.

### 2.6. Statistical Analysis

Statistical analysis was conducted using IBM SPSS Statistics software 26.0 (IBM Corp, Armonk, NY, USA), Microsoft Excel 2016 (Microsoft Corporation, Redmond, WA, USA) and RStudio version 4.0.2 (Boston, MA, USA). Descriptive statistics are presented as absolute numbers and percentages for categorical variables and as either mean and 95%CI or median and IQR for continuous variables

## 3. Results

### 3.1. Patient Characteristics

During our study period, 252 COVID-19 patients were admitted to the ICU. Median age was 57 (IQR: 46–65) and 32.1% were female. Most of the patients were mechanically ventilated (202 of 252; 80.2%) and nearly a third died during their ICU stay (76 of 252; 30.2%). Median length of stay at ICU was 26 days (IQR: 11.8–41.3). Further characteristics are shown in Table 2.

Out of those 252 COVID-19 patients at the ICU, 172 were screened for fungal infection using the β-D-Glucan assay (68.3%), 163 using the galactomannan assay (64.7%) and 104 using fungal culture from respiratory specimens (41.3%). We identified 36 patients with positive fungal culture, galactomannan assay or β-D-Glucan assay. Of those 36 patients, eleven patients were excluded, not meeting any of the criteria for invasive aspergillosis, e.g., when culture was positive for fungi other than aspergillus or when only β-D-Glucan was positive. Characteristics of the resulting 25 (of 252, 9.9%) patients with IA and COVID-19 are summarized in Table 3; detailed information on diagnostics for each patient is displayed in Table 4.

Out of 25 patients, 17 were male (68%), and the median age was 60 (IQR: 54–68), ranging from 44 to 84 years. The majority of patients were transferred either from another hospital to the ICU (18 of 25; 72%) or from a regular ward at VGH (6 of 25; 24%), and only one patient was admitted directly from home (4%). Diagnosis of COVID-19 was established before admission for all but two patients (23 of 25; 92%). Median SAPS II score on admission was 41 (IQR: 32.5–49), reflecting a 25% probability of in-hospital death [21]. All patients were mechanically ventilated, 16 received ECMO therapy (64%), and 21 (84%) patients received corticosteroids; none of the patients were treated with IL-6 inhibitors. Median length of stay was 28 days (IQR: 21–39), with two patients staying for 80 and 81 days. All but three patients suffered from at least one underlying chronic condition (22 of 25; 88%, Table 3), the most frequent being arterial hypertension (15; 60%), followed by diabetes mellitus (9; 36%). Concerning lung diseases, three patients suffered from COPD (12%), two from bronchial asthma (8%), and one patient had lung cancer, pulmonary emphysema or had undergone lung transplantation, respectively (4%). Further diagnoses included other cardiovascular diseases, depression and hematologic diseases, though most patients did not suffer from any severe disease prior to their SARS-CoV-2 infection, resulting in a non-fatal McCabe score for most of the patients (23 of 25; 92%). The score was ultimately fatal for one patient and rapidly fatal for another (4% each). In total, 76 of 252 (30.2%) COVID-19 ICU patients died within the study period; in patients with COVID-19 and IA, more than half of the patients (14 of 25; 56%) died.

### 3.2. Fungal Infections and Diagnosis

**Figure 2:**
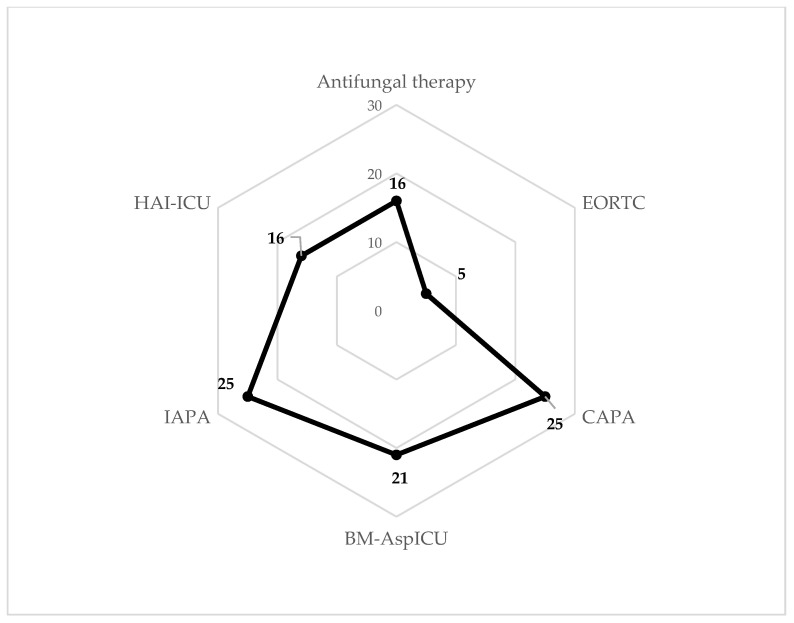
Network diagram of diagnostic criteria. Abbreviations: BM-AspICU = Biomarker-invasive aspergillosis in ICU; CAPA = COVID-19-associated pulmonary aspergillosis; EORTC/MSG = European Organization for the Research and Treatment of Cancer/Mycoses Study Group Education and Research Consortium; HAI-ICU = ECDC HAI-Net ICU protocol, vs. 2.2; IAPA = Influenza-associated pulmonary aspergillosis.

Results for different diagnostic algorithms are displayed in Table 3 and Figure 2, and characteristics of infections in Table 5. Applying ECDC-based routine surveillance criteria for healthcare-associated infections in ICU patients, 16 cases of pulmonary aspergillosis PN3 were found. For EORTC/MSG, BM-AspICU and IAPA 5, 21 and 25 cases of probable IA were detected, respectively. By application of the COVID-19-associated algorithm (CAPA), 23 probable and two possible IA cases were identified. Of the 5 patients detected as probable cases by EORTC/MSG, and of the 21 patients identified by BM-AspICU, 4 and 15 were also classified as PN3, respectively. Both patients with possible IA according to CAPA were also identified by the HAI-ICU protocol as PN3. In total, 17 of 25 included patients (68%) were treated for IA; the majority received azole-antimycotics (15 of 25; 60%), primarily voriconazole (12; 48%). Four patients were treated with echinocandins (16%) and two patients with amphotericin B (8%), the latter one only in combination with azole antimycotics. Five patients received more than one antimycotic agent (20%). Five of the patients receiving antifungal therapy (5 of 17; 29.4%) were not identified as having pulmonary aspergillosis by the HAI-ICU surveillance protocol. Patients with possible IA according to CAPA did not receive antifungal therapy. None of the identified patients ultimately fulfilled the criteria of proven IA as tissue biopsies were not obtained for any of them. For one patient who was classified as a probable case according to IAPA and CAPA, IA was confirmed at autopsy.

**Figure 3:**
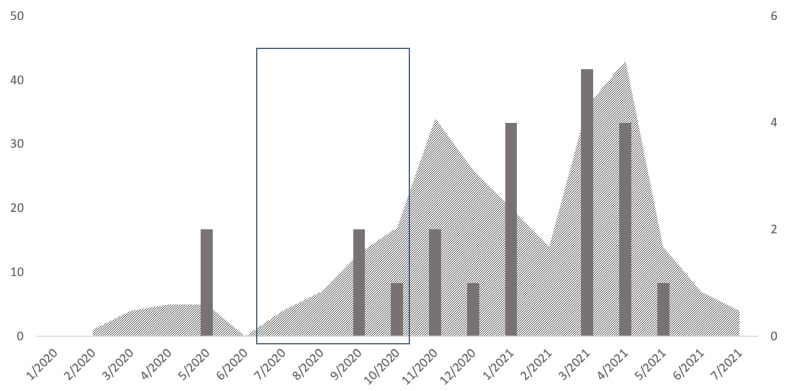
Epidemic curve of COVID-19 cases at ICU (area; y-axis on the left) and COVID-19 plus IA cases at ICU (bars; y-axis on the right); frame gives period of demolition work.

**Figure 4:**
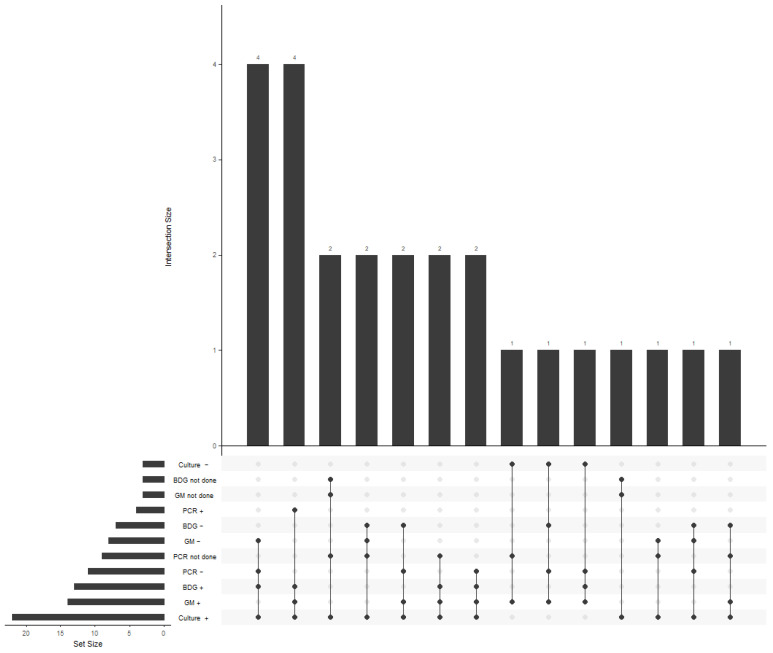
UpSet plot of diagnostic tests. Abbreviations: BDG = β-D-Glucan assay from serum; GM = Galactomannan assay; PCR = Polymerase chain reaction.

For patients with a known start date of COVID-19 infection (*n* = 22), the time from diagnosis of COVID-19 to diagnosis of IA (date of diagnostic sample collection) ranged from seven to 37 days (Median: 18; IQR: 11–26; Table 5). Unsurprisingly, the number of aspergillus-associated co-infections peaked in winter and spring 2021, coinciding with a peak in COVID-19 cases at ICUs in general (Figure 3). All infections were located in the lungs. Most of the infections were diagnosed via cultural methods (22 of 25; 88%); almost half of the culturally diagnosed infections were additionally detected by a positive β-D-Glucan assay (12 of 25; 48%) or a galactomannan assay (11 of 25; 44%); eight were indicated by both antigen assays (of 25; 32%) and four (of 25; 16%) additionally by a positive PCR. The three culture-negative infections were identified by galactomannan assay (3 of 25; 12%), one additionally by both antigen assays (4%). Only one patient had a positive galactomannan assay from serum (4%). Correlation and frequencies of diagnostic results are displayed in an UpSet plot (Figure 4) [22,23]. Culture-positive infections were primarily caused by *Aspergillus fumigatus* (18 of 25; 72%). We did not identify any polymicrobial infections. Resistance testing was performed for 12 isolates (10 *A. fumigatus*, 1 *A. nidulans/terreus*, respectively). All isolates were susceptible to amphotericin B, itraconazole, voriconazole and isavuconazole, except *A. terreus* and *A. nidulans*, which are considered poor targets for amphotericin B. Five out of ten isolates were considered susceptible at increased exposure to posaconazole, while the rest were susceptible.

Of 16 cases classified as PN3, nine died within the observation period (56.3%). Four patients (of 5; 80%) who had probable IA according to EORTC/MSG died, 12 (of 21; 57.1%) according to BM-AspICU. All patients who did not survive the study period were probable cases according to IAPA and CAPA.

## 4. Discussion

Diagnosing IA in ventilated COVID-19 patients is notoriously challenging. Clinical presentation and radiological findings of pulmonary aspergillosis may be very similar to those of COVID-19 ARDS, particularly if CT scans cannot be easily obtained due to the severe clinical condition of these patients. Only 10 of our patients underwent CT scanning, which mostly revealed pulmonary infiltrates that were not pathognomonic for IA. One patient had a cavitary lesion that was attributed to IA and one showed signs that the radiologist described as congruent with fungal pneumonia. Surprisingly, the latter patient did not receive antifungal therapy despite repeated positive aspergillus cultures and galactomannan assays from BAL and was eventually discharged after more than 70 days in the ICU. Clinicians probably suspected *Pseudomonas aeruginosa* as the causative agent for pulmonary superinfection and regarded *Aspergillus fumigatus* as a contaminant.

All other patients had regular chest X-rays at the ICU, where mostly dense infiltrates and ground glass opacities were described. Infiltrates that cannot be attributed to other causes allow classification as probable cases according to IAPA and CAPA as well as HAI-ICU, which may reduce specificity and leaves room for interpersonal differences in interpretation when bacteria are recovered from respiratory specimens as well as aspergillus.

All patients underwent bronchoscopy multiple times in spite of concerns regarding staff safety (especially in the early phase of the pandemic), thus reducing the probability of mere aspergillus colonization of the upper airways, which has to be considered when only sputum or tracheal aspirate can be analyzed. This may largely be due to the fact that 80% of our patients were transferred from other ICUs and may therefore no longer have been considered highly infectious by the time of admission at VGH, which reflects VGH’s role as a referral center for the most severe cases of COVID-19 ARDS.

We were surprised to find that only one out of 23 patients (4.3%) who were screened for GM in serum had a positive result, compared to fifteen out of 23 (65.2%) that were positive for BDG. This corresponds to earlier findings of low sensitivity in immunosuppressed ventilated patients, where the sensitivity of serum GM was 35% compared to 88% for BDG [24], as well as to that in immunocompetent patients, where sensitivity for serum GM was 24.3% [25].

Some experts have expressed concerns of overtreatment with antifungals when applying highly sensitive criteria that are mainly based on mycological evidence [14]. In our patients, five did not receive antifungal treatment even though CAPA criteria of probable IA were fulfilled. Another two patients may be classified as possible cases, but did not receive antifungals either. In our opinion, this indicates that the diagnosis of IA is still made on an individual basis at the discretion of the treating physicians. Retrospectively, it seems that therapy in patients that were probable or possible cases was also guided by BDG as only one out of 15 patients with at least one positive serum BDG result, compared to seven out of eight (87.5%) with negative BDG, did not receive any antifungal therapy.

When comparing the different diagnostic algorithms, influenza-specific (IAPA) and COVID-specific (CAPA) algorithms lead to similar results. Using EORTC/MSG and BM-AspICU criteria, fewer patients were classified as probable cases, due to the lack of immunosuppression in a traditional sense. In any case, cultures from lower respiratory tract specimens remained the main pillar for the presumptive diagnosis of IA. A publication by Fekkar et al. [26] highlights the discrepancy between definitions for IA in COVID-19 patients. The authors retrospectively applied the ECMM ISHAM definitions for CAPA to 17 published cohorts, which brought the overall incidence from 10.9% down to 6.1%. Therefore, a uniform definition of the pathological entity is of the utmost importance in order to estimate the true incidence.

Only seventeen out of 25 probable or possible cases of IA were detected when applying routine surveillance according to the ECDC 2017 protocol, which aims primarily at the detection of infections caused by classical nosocomial pathogens such as *S. aureus*, enterobacterales and nonfermenting gram-negatives. HAI-Net ICU is obviously not designed for the surveillance of fungal infections of the lung, as purulent sputum, fever and leukocytosis may be absent in aspergillosis, but are the prerequisites for the classification of PN. Regarding nosocomial IA acquired at the ICU due to the exceptional circumstances, such as the reversed airflow and demolition work, a more focused surveillance approach will be needed. This is all the more true as the determination of the healthcare association of IA is complicated by prolonged incubation periods, though, in our setting, the minimum duration from in-hospital detection of SARS-CoV-2 until IA was one week, and the median time was 18 days, indicating the nosocomial origin for most of the infections.

Using the most sensitive algorithm, 9.9% (*n* = 25/252) of all COVID-19 patients admitted to VGH’s intensive care units from March 2020 to July 2021 developed IA. This number is in line with data on patients with ARDS due to causes other than COVID-19 [27,28]. It is comparable to data on CAPA from another Austrian center, where incidence was estimated at 10.7% [29], but slightly below published data from international authors who reported rates between 14.1 and 27.7% [7,30,31,32].

Most patients had no severe underlying diseases, reflected by a non-fatal McCabe score in 92%. They rather presented with classical features of metabolic syndrome, such as obesity, non-insulin dependent diabetes mellitus and hypertension, which certainly affect a large proportion of Austrians. This again does not feed into the narrative that only the very old and very sick COVID-19 patients suffer the most severe forms of COVID-19.

Our study has several limitations. Firstly, it is purely descriptive, as we could not identify a suitable control group. COVID-19 is a relatively new entity with unique pathognomonic features; therefore, comparison with other ICU patients is difficult. Patients with acute respiratory distress syndrome due to other underlying illnesses are not stringently screened for IA; therefore, retrospective comparison seemed inappropriate.

Secondly, autopsy was only performed on two of the patients; for all other fatal cases, pathological workup was forgone due to staff safety concerns, the fact that the reasons for demise were conclusive for the treating clinicians and due to relatives’ preferences. This is understandable, but makes a final classification of cases and the assessment of the diagnostic algorithms’ validity in this particular collective difficult. It also impedes the establishment of a causal relationship between fungal infection and the high mortality in our collective. Lastly, when comparing IA rates between institutions, one should exercise caution due to external factors, such as geographical variation or, as in our setting, extensive construction and demolition work in the hospital surroundings.

## 5. Conclusions

In our study, a significant proportion of patients developed IA; most of these patients had no severe underlying disease, but mortality was still comparatively very high. From an infection prevention and control perspective, for the surveillance of IA in a highly vulnerable patient cohort, such as patients with severe COVID-19, applying an algorithm especially designed for respiratory viral illness seems useful. This is particularly true when aggravating circumstances such as reversed airflow and large construction and demolition work are present.

## Figures and Tables

**Table 1:** Main features of diagnostic algorithms.

	HAI-Net ICU PN3	EORTC	BM-AspICU	IAPA	CAPA
Host factors	Admission to ICU > 48 h	Neutropenia Hematologic malignancy Allogeneic stem cell transplant Solid organ transplant Prolonged use of corticosteroids Treatment with T-cell immunosuppressants Treatment with B-cell immunosuppressants Inherited severe immunodeficiency acute GvHD grade III/IV	Risk factors: Neutropenia Hematologic malignancy Allogeneic stem cell transplant Solid organ transplant Prolonged use of corticosteroids Treatment with T-cell immunosuppressants Treatment with B-cell immunosuppressants Inherited severe immunodeficiency Acute GvHD grad III/IV Other risk factors: Chronic obstructive pulmonary disease Viral respiratory diseases (influenza infection, SARS-CoV2 infection, etc.) Cirrhosis, hepatic insufficiency Other (diabetes, chronic alcohol abuse, chronic diseases, cardiac surgery, etc.)	Influenza-like illness, positive influenza PCR or antigen and temporal relationship	Patient with COVID-19 needing intensive care and a temporal relationship
Clinical features	Fever OR leucopenia/leukocytosis AND	Pulmonary aspergillosis: N/A Tracheobronchitis: tracheobronchial ulceration, nodule, pseudomembrane, plaque or eschar	Fever refractory to >3 days of antibiotic therapy Pleuritic chest pain Dyspnea Hemoptysis	Pulmonary aspergillosis: N/A	Pulmonary aspergillosis: refractory fever, pleural rub, chest pain, haemoptysis or a combination
Clinical features	new onset of purulent sputum, or change in character of sputum OR cough/dyspnea/tachypnea OR suggestive auscultation OR worsening gas exchange	in bronchoscopy	Respiratory insufficiency despite ventilation support	Tracheobronchitis: Airway plaque, pseudomembrane or ulcer	Tracheobronchitis: tracheobronchial ulceration, nodule, pseudomembrane, plaque or eschar in bronchoscopy
Radiology	At least 2 chest X-rays or CT scans with suggestive image of pneumonia	Presence of 1 of the following 4 patterns on CT: dense, well-circumscribed lesions with or without a halo sign air crescent sign cavity wedge-shaped and segmental or lobar consolidation	Air-crescent sign Cavity Dense, well-circumscribed lesion(s) with or without halo sign Diffuse reticular and alveolar opacities Nonspecific infiltrates and consolidation Pleural fluid Wedge-shaped infiltrate Tree-in-bud pattern	Pulmonary infiltrate or cavitating infiltrate (not attributed to another cause)	Pulmonary infiltrate (preferably chest CT) or cavitating infiltrate (not attributed to another cause)
Mycological evidence	Positive exam for pneumonia with particular germs (e.g., aspergillus): detection of antigen from respiratory secretions positive direct exam or positive culture from bronchial secretions or tissue	Proven IA: positive histo-/cytopathologic or direct microscopic examination; specimen obtained by needle aspiration or biopsy positive culture from sterile specimen (excluding BAL) positive PCR combined with DNA sequencing when molds are seen in tissue	Proven IA: positive histo-/cytopathologic or direct microscopic examination; specimen obtained by needle aspiration or biopsy positive culture from sterile specimen (excluding BAL) positive PCR combined with DNA sequencing when molds are seen in tissue Probable IA: positive direct examination or aspergillus culture in BAL	Proven IA: biopsy or brush specimen of airway plaque, pseudomembrane, ulcer and positive culture positive PCR in tissue lung biopsy showing invasive fungal elements and Aspergillus growth on	Proven IA: histopathological or direct microscopic detection in tissue positive culture, microscopy, histology or PCR from specimen obtained by sterile aspiration or biopsy Probable IA: positive microscopy or culture in BAL serum galactomannan index > 0.5 serum LFA index > 0.5 bronchoalveolar lavage galactomannan index ≥ 1.0
Mycological evidence		Probable IA: positive culture from sputum, BAL, bronchial brush, aspirate positive microscopy of fungal elements from sputum, BAL, bronchial brush, aspirate galactomannan in single serum or plasma: ≥1.0 galactomannan in BAL fluid: ≥1.0 galactomannan in single serum or plasma: ≥0.7 and BAL fluid ≥ 0.8 at least 2 consecutive PCR tests positive in plasma, serum, whole blood at least 2 duplicate PCR tests positive in BAL at least 1 PCR test positive in plasma, serum or whole blood and 1 PCR test positive in BAL fluid	positive aspergillus culture in lower respiratory tract specimen positive galactomannan in BAL positive aspergillus PCR in BAL positive serum/plasma galactomannan positive serum/plasma aspergillus PCR	culture or positive Aspergillus PCR in tissue Probable IA: positive microscopy serum galactomannan index > 0.5 BAL galactomannan index ≥ 1.0 positive culture from in BAL, non-bronchoscopic lavage, tracheal aspirate or sputum	at least 2 positive aspergillus PCR tests in plasma, serum or whole blood at least 1 positive aspergillus PCR in BAL (<36 cycles) 1 positive aspergillus PCR in plasma, serum, whole blood and 1 positive in bronchoalveolar lavage fluid (any threshold cycle) Possible IA: positive microscopy or culture in non-bronchoscopic lavage single non-bronchoscopic lavage galactomannan index > 4.5 at least 2 non-bronchoscopic lavage galactomannan indices > 1.2 1 non-bronchoscopic lavage galactomannan index > 1.2 plus 1 other positive non-bronchoscopic lavage mycology test

Abbreviations: BAL = Bronchoalveolar lavage; BM-AspICU = Biomarker-invasive aspergillosis in ICU; CAPA = COVID-19-associated pulmonary aspergillosis; COVID-19 = Coronavirus disease 2019; CT = Computed tomography; EORTC/MSG = European Organization for the Research and Treatment of Cancer/Mycoses Study Group Education and Research Consortium; GvHD = Graft-versus-host disease; HAI = Healthcare-associated infection; IA = Invasive aspergillosis; IAPA = Influenza-associated pulmonary aspergillosis; ICU = Intensive care unit; LFA = Lateral flow assay; N/A = Not applicable; PCR = Polymerase chain reaction; PN = Pneumonia.

**Table 2:** Characteristics of all COVID-19 patients at ICU.

	All (*n* = 252)
Age (Median, IQR)	57 (46–65)
Female (%)	81 (32.14%)
Hemoglobin (Mean, SD)	11.03 g/dL (2.13)
Thrombocytes (Median, IQR)	229 g/L (166–301.5)
Leukocytes (Median, IQR)	11.01 g/L (7.82–14.78)
Creatinine (Median, IQR	0.83 mg/dL (0.61–1.29)
CRP (Median, IQR)	12.04 mg/dL (5.36–21.58)
Mechanical ventilation	202 (80.16%)
LOS at ICU in days (Median, IQR)	25.5 (11.75–41.25)
ICU death	76 (30.16%)

Abbreviations: ICU = Intensive care unit; IQR = Interquartile range; LOS = Length of stay; SD = Standard deviation.

**Table 3:** Characteristics of COVID-19 patients at ICU with IA.

	*n* = 25
Female (%)	8 (32%)
Age (Median, IQR)	60 (54–68)
SAPS II score (Median, IQR)	41 (32.5–49)
McCabe score on admission	
Non-fatal	23 (92%)
Ultimately fatal	1 (4%)
Rapidly fatal	1 (4%)
Type of admission	
Direct	1 (4%)
Regular ward in-house	6 (24%)
Another hospital	18 (72%)
LOS ICU in days (Median, IQR)	28 (21–9)
ECMO (%)	16 (64%)
Mechanical ventilation (%)	25 (100%)
ICU death (%)	14 (56%)
COVID-19 on admission (%)	23 (92%)
Therapy with corticosteroids (%)	21 (84%)
Median duration in days (IQR)	10 (2.75–17)
Therapy with IL-6 inhibitors	0 (0%)

Abbreviations: COVID-19 = Coronavirus disease 2019; ECMO = Extracorporeal membrane oxygenation; ICU = Intensive care unit, IQR = Interquartile range; LOS = Length of stay; SAPS = Simplified Acute Physiology Score.

**Table 4:** Results of diagnostics and treatment for each patient.

	Clinical Factors				Mycological Evidence				Diagnostic Codes					Anti-Fungals	ICU Death
	Underlying Diseases (McCabe Score)	Imaging	HAI-ICU	EORTC Host Factors	Culture	Antigen	PCR	Histo-Pathology	HAI-ICU	EORTC	BM-Asp-ICU	IAPA	CAPA		
1	Arterial Hypertension, Diabetes, Steatosis hepatis (NF)	Opacities		-	BAL	GM (Serum) BDG	-	-	-	-	Prob	Prob	Prob	Az	Yes
2	Diabetes, Hypothyreosis (NF)	Small nodules, infiltrates	Leucocytosis	-	BAL	GM (BAL, Serum) BDG	-	-	-	-	Prob	Prob	Prob	Ec	No
3	Arterial Hypertension, Diabetes, Rheumatoid Arthritis (NF)	Ground glass opacities, condensations	Fever, Leucocytosis	Immunosuppressant (Rituximab)	BAL	GM (BAL, Serum) BDG	-	-	PN3	Prob	Prob	Prob	Prob	Az	No
4	Arterial Hypertension, Atrial fibrillation, St. p. N. mammae (NF)	Patchy opacities	Leukopenia		BAL	BDG	-	-	PN3	-	Prob	Prob	Prob	Az	No
5	Arterial Hypertension, Asthma bronchiale (NF)	Ground glass opacities, condensations	Fever, Leucocytosis	-	BAL	GM (BAL) BDG	Fungal broad-spectrum (Blood, tracheal aspirate); *Aspergillus* spp. (BAL, tracheal aspirate)	-	-	-	Prob	Prob	Prob	AmB, Az, Ec	Yes
6	Arterial Hypertension, Hypothyreosis (NF)	Dense infiltrates	Fever, Worsening Gas Exchange	-	BAL	-	-	-	PN3	-	Prob	Prob	Prob	-	No
7	Arterial Hypertension, Depression, Nicotine abuse (NF)	Ground glass opacities	Fever, Leucocytosis	-	BAL	GM BDG	*A. fumigatus* (material not specified)	-	PN3	-	Prob	Prob	Prob	Az	Yes
8	Diabetes (NF)	Condensations, opacities	Fever	-	Tracheal secretion	-	-	-	PN3	-	Prob	Prob	Poss	-	No
9	Arteriitis temporalis, CHF, N. bronchi (UF)	Ground glass opacities, pleural effusion	Leucocytosis	-	BAL	-	-	-	PN3	-	Prob	Prob	Prob	-	Yes
10	CLL, COPD (NF)	Ground glass opacities, condensations	Fever, Leucocytosis	Leukaemia	BAL	BDG	-	-	PN3	Prob	Prob	Prob	Prob	Az	Yes
11	CAOD (St. p. stroke), Diabetes (NF)	Nodular lesions, condensations, pleural effusions	-	-	BAL	GM (BAL)	-	-	-	-	Prob	Prob	Prob	-	Yes
12	- (NF)	Ground glass opacities, nodular condensations	Fever, Leucocytosis	-	-	GM (BAL)	-	-	-	-	-	Prob	Prob	Az	No
13	Arterial Hypertension, Diabetes, PAOD, Nicotin abuse (NF)	Dense condensations, pleural effusions	-	-	BAL	-	-	-	-	-	-	Prob	Prob	-	Yes
14	Arterial Hypertension, Atrial fibrillation, COPD, Diabetes (NF)	Ground glass opacities, pleural effusions	Leucocytosis	-	BAL	BDG	-	-	PN3	-	-	Prob	Prob	Ec	Yes
15	Lymphoma (NF)	Dense opacities	-	Lymphoma	Tracheal secretion	GM (BAL) BDG	-	-	-	Prob	Prob	Prob	Prob	Az	Yes
16	Arterial Hypertension, Asthma bronchiale (NF)	Patchy opacities	Leucocytosis	-	BAL	GM (BAL) BDG	*A. fumigatus* (BAL)	-	PN3	-	Prob	Prob	Prob	Az, Ec	Yes
17	Arterial Hypertension, Asthma bronchiale, Obesity (NF)	Nodular opacities	Leucocytosis.	-	Bronchial secretion	-	-	-	PN3	-	-	Prob	Poss	-	No
18	Arterial Hypertension, CHD, Diabetes (NF)	Ground glass opacities, condensations, bullae	Leucocytosis, Worsening Gas Exchange, Purulent Sputum	Immunosuppressant (Corticosteroids)	BAL	-	-	-	PN3	Prob	Prob	Prob	Prob	Az	Yes
19	Arterial Hypertension, CKD, COPD (NF)	Ground glass opacities, condensations, dystelectasis	Fever, Leucocytosis, Worsening Gas Exchange	-	-	GM (BAL)	-	-	PN3	-	Prob	Prob	Prob	Az	Yes
20	Arterial Hypertension, CHD, Diabetes, Sleep apnea (NF)	Dense opacities, white lung	Leucocytosis	-	BAL	-	-	-	PN3	-	Prob	Prob	Prob	Az	Yes
21	Arterial Hypertension, Depression (NF)	Left complete atelectasis, dense opacities	Leucocytosis	-	BAL	GM (BAL)BDG	*A. fumigatus* (material not specified)	-	PN3	-	Prob	Prob	Prob	AmB, Az	No
22	End-stage lymphoma, Pulmonary Emphysema, Nicotine abuse (RF)	Ground glass opacities, condensations	Fever, Leucocytosis	Lymphoma	BAL	BDG	-	Aspergillus in autopsy	PN3	Prob	Prob	Prob	Prob	Az	Yes
23	-(NF)	Cavitary lesion, dense opacities	-	-	BAL	GM (BAL)	-	-	-	-	Prob	Prob	Prob	-	Yes
24	St. p. Hepatitis C (NF)	Patchy condensations, ground glass opacities	-	-	BAL	GM (BAL)	-	-	-	-	Prob	Prob	Prob	-	No
25	Arterial Hypertension (NF)	Nodular condensations	Leucocytosis, Worsening Gas Exchange, Purulent Sputum	-	-	GM (BAL)	-	-	PN3	-	Prob	Prob	Prob	Az	No

Abbreviations: AmB = Amphotericin B; Az = Azoles; BAL = Bronchoalveolar lavage; β-D = β-D-Glucan assay from serum; BM-AspICU = Biomarker-invasive aspergillosis in ICU; CAOD = Cerebral arterial occlusive disease; CAPA = COVID-19-associated pulmonary aspergillosis; CHD = Coronary heart disease; CKD = Chronic kidney disease; CLL = Chronic lymphocytic leukemia; COPD = Chronic obstructive pulmonary disease; Ec = Echinocandins; EORTC/MSG = European Organization for the Research and Treatment of Cancer/Mycoses Study Group Education and Research Consortium; GM = Galactomannan assay; HAI-ICU = HAI-ICU = ECDC HAI-Net ICU protocol, vs. 2.2; IAPA = Influenza-associated pulmonary aspergillosis; N. = Neoplasia; NF = Non-fatal; PAOD = Peripheral arterial occlusive disease; PN = Pneumonia; Poss = Possible; Prob = Probable; RF = Rapidly fatal; SOT = Solid organ transplantation; St. p. = Status post; UF = Ultimately fatal.

**Table 5:** Characteristics IA.

	*n* = 25
Fungal infection on admission (%)	7 (28%)
Median time from COVID-19 to fungal infection in days (IQR) (*n* = 22)	18 (11–26)
Diagnostics	
Culture	22 (88%)
Galactomannan assay from serum or BAL	14 (56%)
β-D-Glucan assay from serum or BAL	13 (52%)
PCR	4 (16%)
Fungal species	
*Aspergillus fumigatus*	18 (69.2%)
*Aspergillus flavus*	1 (4%)
*Aspergillus fumigatiaffinus*	1 (4%)
*Aspergillus nidulans*	1 (4%)
*Aspergillus terreus*	1 (4%))
More than one	0 (0%)
No cultural growth	3 (12%)
Organ affected	
Lung	25 (100%)
Therapy with antifungal agents ^a^	17 (68%)
Azoles	15 (60%)
Voriconazole	12 (48%)
Isavuconazole	3 (12%)
Fluconazole	1 (4%)
Posaconazole	1 (4%)
Echinocandins	5 (20%)
Anidulafungin	2 (8%)
Caspofungin	2 (8%)
Micafungin	1 (4%)
Amphotericin B	2 (8%)
Death within study period	14 (56%)

^a^ Percentages may not add up to 100% due to therapy with multiple agents. Abbreviations: BAL = Broncheoalveolar lavage; IA = Invasive aspergillosis; IQR = Interquartile range; PCR = Polymerase chain reaction.

## Data Availability

Data not available on request due to restrictions.

## Supplementary Materials

The following supporting information can be downloaded at: https://www.mdpi.com/article/10.3390/jof8030273/s1, Supplement File S1: Diagnostic algorithms.
