# Supplementary material for: Routine Surveillance of Healthcare-Associated Infections Misses a Significant Proportion of Invasive Aspergillosis in Patients with Severe COVID-19
Source: J Fungi (Basel). 2022 Mar 8;8(3):273. doi: 10.3390/jof8030273 (PMC8954197; doi:10.3390/jof8030273)
Supplement: Supplementary file 1 [file jof-08-00273-s001.zip › jof-1611780-supplementary.pdf]

Supplementary File S1. Diagnostic algorithms

1. **HAI-ICU**

X-ray

Two or more serial chest X-rays or CT-scans with a suggestive image of pneumonia for patients with underlying cardiac or pulmonary disease* (in patients without underlying cardiac or pulmonary disease, one definitive chest X-ray or CT-scan is sufficient).

Symptoms

and at least one of the following:

- fever > 38 °C with no other cause
- leukopenia (< 4 000 WBC/mm^3) or leucocytosis (≥ 12 000 WBC/mm^3).

and at least one of the following (or at least two, if clinical pneumonia only = PN 4 and PN 5):

- new onset of purulent sputum, or change in character of sputum (colour, odour, quantity, consistency)
- cough or dyspnoea or tachypnea
- suggestive auscultation (rales or bronchial breath sounds), rhonchi, wheezing
- worsening gas exchange (e.g. O_2 desaturation or increased oxygen requirements or increased ventilation demand)

and according to the used diagnostic method:

Microbiology

a) Bacteriologic diagnostic performed by:

Positive quantitative culture from minimally contaminated LRT specimen (**PN 1**)

- bronchoalveolar lavage (BAL) with a threshold of ≥ 10^4 colony forming units (CFU)/ml or ≥ 5% of BAL obtained cells contain intracellular bacteria on direct microscopic exam (classified on the diagnostic category BAL)
- protected brush (PB Wimberley) with a threshold of ≥ 10^3 CFU/ml
- distal protected aspirate (DPA) with a threshold of ≥ 10^3 CFU/ml.

Positive quantitative culture from possibly contaminated LRT specimen (**PN 2**)

- Quantitative culture of LRT specimen (e.g. endotracheal aspirate) with a threshold of 10^6 CFU/ml.

b) Alternative microbiology methods (**PN 3**)

- positive blood culture not related to another source of infection
- positive growth in culture of pleural fluid
- pleural or pulmonary abscess with positive needle aspiration
- histologic pulmonary exam shows evidence of pneumonia

- positive exams for pneumonia with virus or particular germs (e.g. Legionella, Aspergillus, mycobacteria, mycoplasma, *Pneumocystis jiroveci* [previously *P. carinii*]):
- positive detection of viral antigen or antibody from respiratory secretions (e.g. EIA, FAMA, shell vial assay, PCR)
- positive direct exam or positive culture from bronchial secretions or tissue
- seroconversion (example: influenza viruses, Legionella, Chlamydia)
- detection of antigens in urine (Legionella).

c) Others

- positive sputum culture or non-quantitative LRT specimen culture (**PN 4**)
- no positive microbiology (**PN 5**).

2. **EORTC/MSG**

Proven

Microscopic Analysis: Sterile Material: Histopathologic, cytopathologic, or direct microscopic examination of a specimen obtained by needle aspiration or biopsy in which hyphae or melanized yeast-like forms are seen accompanied by evidence of associated tissue damage

or

Culture: Sterile Material: Recovery of a hyaline or pigmented mold by culture of a specimen obtained by a sterile procedure from a normally sterile and clinically or radiologically abnormal site consistent with an infectious disease process, excluding BAL fluid, a paranasal or mastoid sinus cavity specimen, and urine

or

Blood: Blood culture that yields a mold (eg, Fusarium species) in the context of a compatible infectious disease process

or

Tissue Nucleic Acid Diagnosis: Amplification of fungal DNA by PCR combined with DNA sequencing when molds are seen in formalin-fixed paraffin-embedded tissue

Probable

At least one of the following host factors

- Recent history of neutropenia (<0.5 × 109 neutrophils/L [<500 neutrophils/mm^3] for >10 days) temporally related to the onset of invasive fungal disease
- Hematologic malignancy
- Receipt of an allogeneic stem cell transplant
- Receipt of a solid organ transplant

- Prolonged use of corticosteroids (excluding among patients with allergic bronchopulmonary aspergillosis) at a therapeutic dose of ≥0.3 mg/kg corticosteroids for ≥3 weeks in the past 60 days
- Treatment with other recognized T-cell immunosuppressants, such as calcineurin inhibitors, tumor necrosis factor-a blockers, lymphocytespecific monoclonal antibodies, immunosuppressive nucleoside analogues during the past 90 days
- Treatment with recognized B-cell immunosuppressants, such as Bruton's tyrosine kinase inhibitors, eg, ibrutinib
- Inherited severe immunodeficiency (such as chronic granulomatous disease, STAT 3 deficiency, or severe combined immunodeficiency)
- Acute graft-versus-host disease grade III or IV involving the gut, lungs, or liver that is refractory to first-line treatment with steroids

<u>At least one of the following clinical features</u>

Pulmonary aspergillosis

One of the following patterns on CT:

- Dense, well-circumscribed lesions(s) with or without a halo sign
- Air crescent sign
- Cavity
- Wedge-shaped and segmental or lobar consolidation

Other pulmonary mold diseases

As for pulmonary aspergillosis but also including a reverse halo sign

Tracheobronchitis

- Tracheobronchial ulceration, nodule, pseudomembrane, plaque, or eschar seen on bronchoscopic analysis

Sino-nasal diseases

- Acute localized pain (including pain radiating to the eye)
- Nasal ulcer with black eschar
- Extension from the paranasal sinus across bony barriers, including into the orbit

Central nervous system infection

One of the following signs:

- Focal lesions on imaging
- Meningeal enhancement on magnetic resonance imaging or CT

<u>At least one of the following mycological evidence</u>

Any mold, for example, Aspergillus, Fusarium, Scedosporium species or Mucorales recovered by culture from sputum, BAL, bronchial brush, or aspirate

Microscopical detection of fungal elements in sputum, BAL, bronchial brush, or aspirate indicating a mold

Tracheobronchitis

- Aspergillus recovered by culture of BAL or bronchial brush
- Microscopic detection of fungal elements in BAL or bronchial brush indicating a mold

Sino-nasal diseases

- Mold recovered by culture of sinus aspirate samples
- Microscopic detection of fungal elements in sinus aspirate samples indicating a mold

Aspergillosis only

Galactomannan antigen detected in plasma, serum, BAL, or CSF of any of the following thresholds:

- Single serum or plasma: ≥1.0
- BAL fluid: ≥1.0
- Single serum or plasma: ≥0.7 and BAL fluid ≥0.8

3. **BM-AspICU**

Entry criterion: admittance to ICU and one of the following

- Positive Aspergillus in the lower respiratory tract
- Imaging sign (CT or X-Ray)
    - Air-crescent sign
    - Cavity
    - Dense, well-circumscribed lesion(s) with or without halo sign
    - Diffuse reticular and alveolar opacities
    - Nonspecific infiltrates and consolidation
    - Pleural fluid
    - Wedge-shaped infiltrate
    - Tree-in-bud pattern
- Clinical sign
    - Fever refractory to > 3 days of antibiotherapy
    - Pleuritic chest pain
    - Dyspnoea
    - Hemoptysis
    - Respiratory insufficiency despite ventilation support

Proven see EORTC

Probable

At least one EORTC/MSG host factor (see above) and at least one imaging sign (see above) and at least one of the following mycological signs

- Positive direct examination showing hyphae

- Positive Aspergillus culture in BALF
- Positive Aspergillus culture in lower respiratory tract specimen
- Fungal biomarkers
- BALF galactomannan
- BALF Aspergillus qPCR
- Serum/plasma galactomannan
- Serum/plasma Aspergillus qPCR

or

at least one of the following:

- Chronic obstructive pulmonary disease
- Viral respiratory diseases (influenza infection, SARS-CoV2 infection, etc.)
- Cirrhosis, hepatic insufficiency
- Other (diabetes, chronic alcohol abuse, chronic diseases, cardiac surgery, etc.)

and at least one imaging sign and at least two mycological signs and at least one clinical sign (see above)

Possible

At least one EORTC/MSG host factor and at least one clinical sign.

4. **<u>IAPA</u>**

Entry criteria: influenza-like illness + positive influenza PCR or antigen + temporally relationship

<u>Aspergillus tracheobronchitis</u>

Proven Biopsy or brush specimen of airway plaque, pseudomembrane or ulcer showing hyphal elements and Aspergillus growth on culture or positive Aspergillus PCR in tissue

Probable Airway plaque, pseudomembrane or ulcer

and at least one of the following:

- Serum GM index > 0.5
- BAL GM index ≥ 1.0
- Positive BAL culture
- Positive tracheal aspirate culture
- Positive sputum culture
- Hyphae consistent with Aspergillus

<u>IAPA in patients without documented Aspergillus tracheobronchitis</u>

Proven Lung biopsy showing invasive fungal elements and Aspergillus growth on culture or positive Aspergillus PCR in tissue

Probable A: Pulmonary infiltrate

and at least one of the following:

- Serum GM index > 0.5
- BAL GM index ≥ 1.0
- Positive BAL culture

or

B: Cavitating infiltrate (not attributed to another cause)

and at least one of the following:

- Positive sputum culture
- Positive tracheal aspirate culture

5. **<u>CAPA</u>**

Entry criterion: Patient with COVID-19 needing intensive care and a temporal relationship

Proven Tracheobronchitis or other pulmonary form

and at least one of the following:

- histopathological or direct microscopic detection of fungal hyphae, showing invasive growth with associated tissue damage
- aspergillus recovered by culture
- microscopy or histology or PCR obtained by a sterile aspiration or biopsy from a pulmonary site, showing an infectious disease process

Probable

<u>Tracheobronchitis</u>

tracheobronchial ulceration, nodule, pseudomembrane, plaque, or eschar seen on bronchoscopic analysis

and at least one of the following: microscopic

- detection of fungal elements in bronchoalveolar lavage, indicating a mould
- positive bronchoalveolar lavage culture or PCR
- serum galactomannan index >0·5
- serum LFA index >0.5
- bronchoalveolar lavage galactomannan index ≥1.0
- bronchoalveolar lavage LFA index ≥1.0

<u>Other pulmonary forms</u>

Pulmonary infiltrate, preferably documented by chest CT, or cavitating infiltrate (not attributed to another cause)

and at least one of the following:

- Microscopic detection of fungal elements in bronchoalveolar lavage, indicating a mould
- positive bronchoalveolar lavage culture

- serum galactomannan index >0.5
- serum LFA index >0.5
- bronchoalveolar lavage galactomannan index ≥1.0
- bronchoalveolar lavage LFA index ≥1.0
- two or more positive aspergillus PCR tests in plasma, serum, or whole blood
- a single positive aspergillus PCR in bronchoalveolar lavage fluid (<36 cycles)
- a single positive aspergillus PCR in plasma, serum, or whole blood, and a single positive in bronchoalveolar lavage fluid (any threshold cycle permitted)

Possible Pulmonary infiltrate, preferably documented by chest CT, or cavitating infiltrate (not attributed to another cause)

and at least one of the following:

- microscopic detection of fungal elements in non-bronchoscopic lavage indicating a mould
- positive non-bronchoscopic lavage culture
- single non-bronchoscopic lavage galactomannan index >4.5
- non-bronchoscopic lavage galactomannan index >1.2 twice or more
- non-bronchoscopic lavage galactomannan index >1.2 plus another non-bronchoscopic lavage mycology test positive (non-bronchoscopic lavage PCR or LFA)
